# Risperidone Decreases Expression of Serotonin Receptor-2A (5-HT2A) and Serotonin Transporter (SERT) but Not Dopamine Receptors and Dopamine Transporter (DAT) in PBMCs from Patients with Schizophrenia

**DOI:** 10.3390/ph17020167

**Published:** 2024-01-28

**Authors:** Samantha Alvarez-Herrera, Mauricio Rosel Vales, Gilberto Pérez-Sánchez, Enrique Becerril-Villanueva, Yvonne Flores-Medina, José Luis Maldonado-García, Ricardo Saracco-Alvarez, Raúl Escamilla, Lenin Pavón

**Affiliations:** 1 Laboratorio de Psicoinmunología, Dirección de Investigaciones en Neurociencias, Instituto Nacional de Psiquiatría Ramón de la Fuente Muñíz, Mexico City 14370, Mexico; dra.alvarezherrera@gmail.com (S.A.-H.); gilberto.perez.sanchez@inprf.gob.mx (G.P.-S.); lusenbeve@inprf.gob.mx (E.B.-V.); 2 Clínica de Esquizofrenia, Dirección de Servicios Clínicos, Instituto Nacional de Psiquiatría Ramón de la Fuente Muñíz, Mexico City 14370, Mexico; m.rosel@imp.edu.mx; 3 Subdirección de Investigaciones Clínicas, Instituto Nacional de Psiquiatría Ramón de la Fuente Muñíz, Mexico City 14370, Mexico; yg.floresmedina@gmail.com (Y.F.-M.); saracco@inpfr.gob.mx (R.S.-A.); 4 Departamemto de Inmunología, Escuela Nacional de Ciencias Biológicas, Instituto Politécnico Nacional, Mexico City 11340, Mexico; joselmgarci@comunidad.unam.mx; 5 Departamemto de Bioquímica, Facultad de Medicina, Universidad Nacional Autónoma de México, Mexico City 04510, Mexico; 6 Subdirección de Consulta Externa, Instituto Nacional de Psiquiatría Ramón de la Fuente Muñíz, Mexico City 14370, Mexico; jefsub@imp.edu.mx

**Keywords:** schizophrenia, dopamine receptors, serotonin receptors, dopamine transporter, serotonin transporter, risperidone

## Abstract

Dopamine and serotonin receptors and transporters play an essential role in the pathophysiology of schizophrenia; changes in their expression have been reported in neurons and leukocytes. Each antipsychotic induces a unique pattern in leukocyte function and phenotype. However, the use of polytherapy to treat schizophrenia makes it challenging to determine the specific effects of risperidone on peripheral blood mononuclear cells (PBMCs). The aim of this study was to evaluate the changes in the expression of D_3_, D_5_, DAT, 5-HT_2A_, and SERT in PBMCs from healthy volunteers (HV), drug-naive patients with schizophrenia (PWS), drug-free PWS, and PWS treated with risperidone for up to 40 weeks using quantitative PCR. Our study revealed elevated mRNA levels of D_3_, DAT, 5-HT_2A_, and SERT in unmedicated PWS. Treatment with risperidone led to a reduction only in the expression of 5-HT_2A_ and SERT. Furthermore, we observed a moderate correlation between 5-HT_2A_ expression and the positive and negative syndrome scale (PANSS), as well as SERT expression and PANSS scale. We also found a moderate correlation between 5-HT_2A_ and SERT expression and the positive subscale. The duration of risperidone consumption had a significant negative correlation with the expression of 5-HT_2A_ and SERT. Our study introduces the measurement of 5-HT_2A_ and SERT expression in PBMCs as a useful parameter for assessing the response to risperidone in PWS.

## 1. Introduction

Schizophrenia is a highly complex and costly neuropsychiatric condition that causes significant impairment both to patients and their families, as well as to society at large [1]. This disease is characterized by a range of symptoms, such as hallucinations, delusions, disorganized thinking, and impairment in language and social or occupational functioning [2]. This disorder has a global prevalence of 1% [3] and patients with schizophrenia (PWS) typically have a lifespan 20 years shorter than that of the overall population, mainly due to issues like cardiovascular disease and suicide [4]. 

The symptomatology of schizophrenia is related to dysfunctional dopaminergic and serotonergic systems in the central nervous system (CNS) [5]. PWS show structural and cellular alterations to the CNS, including changes in the function and expression of dopamine (DA) and serotonin (5-HT) receptors and transporters within various brain regions during acute episodes [6,7]. In the same way, it is well documented that antipsychotics also induce functional changes in neuronal cells, such as alterations in the density of DA and 5-HT receptors and transporters in brain areas [8]. Therefore, neurotransmitter tone and the function and density of receptors in specific brain structures are critical to the condition’s pathophysiology and pharmacotherapy’s efficacy [9] since these receptors are the main therapeutic targets for antipsychotic drugs [10]. 

Various cell types outside the CNS express functional DA and 5-HT receptors and transporters in the cell surface, allowing the cell to respond by sensing its target molecules and their analogs [11]. In particular, leukocytes express dopamine receptor-1 through -D5 (D_1_, D_2_, D_3_, D_4_, D_5_) and serotonin receptor-1 through-4 (5-HT_1,_ 5-HT_2_, 5-HT_3_, 5-HT_4_), -6 (5-HT_6_), -7 (5-HT_7_), in addition to the dopamine transporter (DAT) and serotonin transporter (SERT) in a manner that depends on cell type and activation state [12,13]. Like neuronal cells, leukocytes of PWS display alterations in gene expression of DA and 5-HT receptors and transporters. Notably, the expression of DA receptors is the most extensively studied in leukocytes. However, findings are inconsistent, and the results range from high mRNA levels to lower levels or no changes in patients compared to controls [14]. Unfortunately, 5-HT receptors and SERT in leukocytes are poorly explored, despite their importance in the psychopathology of schizophrenia and the action mechanism of antipsychotic drugs.

Some reports suggest that the expression of DA and 5-HT receptors and transporters in leukocytes reflects the state of receptors in neural cells [15], although this idea is controversial [16]. The evidence shows that leukocytes from PWS exhibit alterations in phenotype and cellular function associated with the occurrence of the disorder or the use of antipsychotic drugs, but this information is not fully clear [17,18]. In addition, research has shown a correlation between the mRNA levels of DA and 5-HT receptors and transporters in leukocytes and the severity of clinical symptoms [14].

Antipsychotics are the principal drugs for both treating psychotic episodes and maintenance pharmacotherapy [19]. While they have been demonstrated to be effective in relieving positive symptoms, their efficacy in improving other symptoms remains unproven [20]. These drugs primarily function as agonists or antagonists of DA and 5-HT receptors, with each drug exhibiting a specific affinity for specific receptors [18]. Additionally, antipsychotics have been found to induce changes in leukocytes, specifically in peripheral blood mononuclear cells (PBMCs) [21,22]. The direct and sustained effects on gene expression in blood cells caused by antipsychotics are evident even after suspending treatment [23].

Antipsychotic drugs activate and modify different signal transduction pathways in PBMCs. Each antipsychotic has a distinct pattern in regulating cellular function [24]. These drugs also modify the expression of DA and 5-HT receptors and transporters in leukocytes [25,26,27]. Various reports have evaluated gene expression in PBMCs of medicated patients; however, the heterogeneity in the treatment of the study groups does not allow the evaluation of the distinct effect each antipsychotic has on blood cell function. 

This study constitutes the initial approach to assess the mRNA levels of D_3_, D_5_, DAT, 5-HT_2A_, and SERT in PBMCs from healthy volunteers (HV), drug-naïve PWS, drug-free PWS, and patients treated from 1 to 40 weeks with risperidone. In addition, we evaluated the severity of patient’s symptoms using the positive and negative syndrome scale (PANSS). Finally, we evaluated the effect of risperidone on the expression of these receptors and their correlation with clinimetric parameters or the duration of risperidone consumption. 

## 2. Results

### 2.1. Participants, Demographic Data, and Scales Scores

The demographic data of drug naïve (DN = 11), drug free (DF = 17), patients treated with risperidone (R = 28), and healthy volunteers (HV = 10) are shown in Table 1. The statistical analysis showed no differences in the body mass index (BMI) or age among groups; the statistical analysis of clinimetric data showed differences in the PANSS total scores (F = 7.652, dƒ = 2, 53; *p* < 0.0012), as well as differences in the scores of the positive (F = 17.41, dƒ = 2, 53; *p* < 0.0001), cognitive (F = 3.296, dƒ = 2,53; *p* < 0.0447), and excitability (H(2) = 6.791; *p* < 0.0335) subscales (Table 1). When we compared the scores between groups, the data showed that drug-naïve patients showed a significant difference in positive subscale compared with patients treated with risperidone (*p* < 0.0006). On the other hand, drug-free patients showed significant differences in PANSS total (*p* < 0.0009), positive (*p* < 0.0001), cognitive (*p* < 0.0364), and excitability (*p* < 0.0287) subscale scores compared with medicated patients. 

### 2.2. Differences in Expression of DA Receptors between Patient and Healthy Volunteers

**Figure 1:**
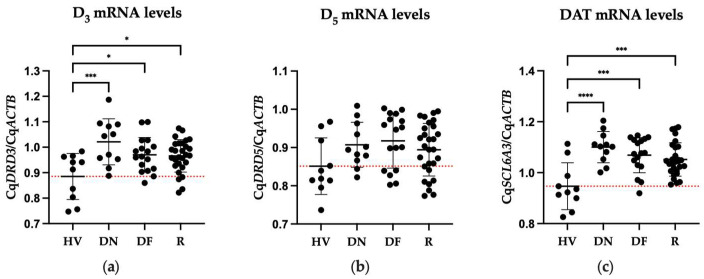
Comparison of mRNA levels of dopamine receptors and transporter of healthy volunteers (HV), drug-naïve (DN), drug-free patients (DF), and patients treated with risperidone (R). Mean ± SD. (**a**) D_3_ mRNA levels; (**b**) D_5_ mRNA levels; (**c**) DAT mRNA levels. The dotted red line shows the mean of the HV group. * = *p* < 0.05, *** = *p* < 0.001 and **** = *p* < 0.0001.

The one-way ANOVA test showed significant differences in mRNA levels of D_3_ (F = 6.037, dƒ = 3, 62; *p* < 0.0011) and DAT (F = 9.488, dƒ = 3, 62; *p* < 0.0001) among the different clinical conditions analyzed (Figure 1). The comparison among groups showed that drug-naïve, drug-free, and patients treated with risperidone presented a significant increase in mRNA levels of D_3_ (DN, *p* < 0.0005; DF, *p* < 0.0264; R, *p* < 0.0217) and DAT (DN, *p* < 0.0001; DF, *p* < 0.0003; R, *p* < 0.0008) compared to HV. 

### 2.3. Differences in Gene Expression of 5-HT_2A_ and SERT of Patients and Healthy Volunteers

**Figure 2:**
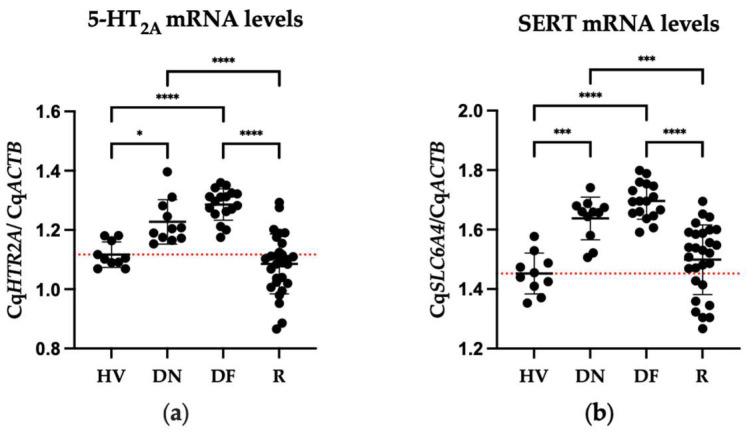
Comparison of mRNA levels of serotonin receptors and transporter of healthy volunteers (HV), drug-naïve (DN), drug-free patients (DF), and patients treated with risperidone (R). Mean ± SD. (**a**) 5-HT_2A_ mRNA levels; (**b**) SERT mRNA levels. The dotted red line shows the mean of the HV group. * = *p* < 0.05, *** = *p* < 0.001 and **** = *p* < 0.0001.

The ANOVA results evidenced that there were statistical differences in the mRNA levels of 5-HT_2A_ (F = 25.77, dƒ = 3, 62; *p* < 0.0001) and SERT (F = 23.68, dƒ = 3, 62; *p* < 0.0001) among groups (Figure 2). Post hoc test showed a significant increase in 5-HT_2A_ mRNA levels in drug-naïve and drug-free patients compared to HV (DN, *p* < 0.0122; DF, *p* < 0.0001). Patients treated with risperidone exhibited a significant decrease in gene expression compared to drug-naïve (*p* < 0.0001) and drug-free (*p* < 0.0001) groups. The SERT expression in drug-naïve and drug-free patients was significantly elevated compared to HV (DN, *p* < 0.0001; DF, *p* < 0.0001). When we compared SERT expression in patients treated with risperidone to those who were not medicated (drug-naïve or drug-free patients), we found a significant decrease in gene expression compared to both non-medicated groups (DN, *p* < 0.0004; DF, *p* < 0.0001). 

### 2.4. Correlation between Gene Expression and Both PANSS Total Score or Subscale Scores, and Duration of Risperidone Consumption

The evaluation of a correlation between the symptomatology and the gene expression of the receptors and transporters showed that only 5-HT_2A_ and SERT exhibited a significant correlation (Table 2). 

**Figure 3:**
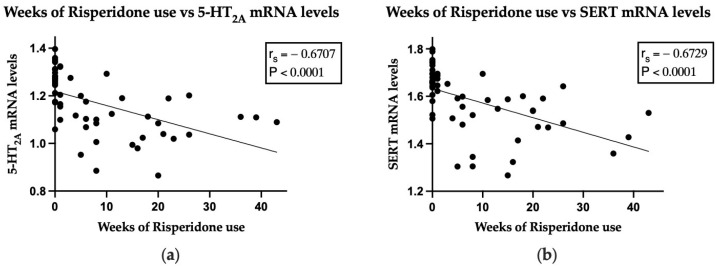
Correlations between weeks of risperidone consumption vs. mRNA levels of 5-HT_2A_ or SERT of patients with schizophrenia. (**a**) Weeks of risperidone use vs. 5-HT_2A_ mRNA levels; (**b**) weeks of risperidone use vs. SERT mRNA levels. Spearman’s rank correlation test.

The mRNA levels of 5-HT_2A_ correlated moderately with both the PANSS total scale (r_s_ = 0.5801, *p* < 0.0001) and the positive subscale (r = 0.5608, *p* < 0.0001). Similarly, the mRNA levels of SERT showed a moderate correlation with the PANSS total scale (r_s_ = 0.5199, *p* < 0.0001), and the positive subscale (r_s_ = 0.5698, *p* < 0.0001). When we analyzed the association between the gene expression of receptors and transporters and the time of risperidone consumption (weeks), the analysis showed a strong inverse correlation only with the mRNA levels of 5-HT_2A_ (r_s_ = −0.6706, *p* < 0.0001) and SERT (r_s_ = −0.6729, *p* < 0.0001). These results suggest that risperidone consumption is associated with a decrease in the mRNA levels of 5-HT_2A_ and SERT, with low mRNA levels being in proportion to a decrease in the positive subscale score, and consequently, to the decrease in the PANSS total scores (Figure 3).

## 3. Discussion

Changes in the density or expression of DA and 5-HT receptors and transporters in the CNS of PWS have been demonstrated by several studies (Table 3); however, there is also experimental evidence of alterations in these molecules in leukocytes associated with the pathophysiology of schizophrenia [14]. Some of these alterations have even been proposed as potential biomarkers for diagnosis or clinical follow up of patients [15]. The altered expression of these molecules in leukocytes may lead to alterations in cellular function that may affect the optimal immune response.

Antipsychotics are the main drugs used to treat schizophrenia [61]. Ideally, the primary targets of these drugs are the receptors of DA and 5-HT in the CNS. However, antipsychotics can also stimulate the receptors expressed on leukocytes; each antipsychotic regulates different features of the immune system, for example, in vitro risperidone and clozapine inhibit Th1 differentiation by suppressing T-bet in T cells, while haloperidol suppresses GATA-3 expression, probably by inhibiting NF-κB activation, thereby inhibiting Th2 differentiation [24]. Regarding the impact of different antipsychotic drugs on PBMCs, the effect may vary depending on the type of receptors to which each antipsychotic has an affinity. Antipsychotic drugs are classified into typical antipsychotics, which have an affinity for dopamine receptors, particularly D_2_, and atypical antipsychotics, which have an affinity for other receptor types such as serotoninergic, glutamatergic, or muscarinic [62]. Specialized reports suggest [24,63] that the biological effect induced by antipsychotic drugs will be more similar when these belong to the same classification, and the effects will be significantly different when comparing the effects of typical vs. atypical antipsychotics.

Several reports have shown that antipsychotics modify the expression of DA and 5-HT receptors and transporters in leukocytes [14], however, the specific effect of each drug on these cells is not well described. Most studies analyze only one group of medicated patients, regardless of whether patients are taking different antipsychotics, although there are few studies that include a group of patients taking a specific drug. 

Risperidone is included in the World Health Organization’s list of essential medicines due to its efficacy, safety, tolerability, and cost effectiveness. It is the most widely prescribed atypical antipsychotic worldwide. Its mechanism of action is associated with a strong affinity for 5-HT_2A_ and antagonism of the D_2_ receptor [18]. Therefore, treatment guidelines recommend it as the first-line choice for treating schizophrenia [64]. The drug’s versatility in various forms of presentation and its described characteristics make it a preferred choice for prescribing patterns in the schizophrenia clinic at the Instituto Nacional de Psiquiatría Ramón de la Fuente Muñiz [65]. These facts support the choice of this drug as the first antipsychotic to initiate our study. 

We chose the two receptors with the highest affinity to dopamine according to published reports. D_3_ is the DA receptor with the highest affinity, and D_5_ is the second (D_3_, K_i_ ≈ 27 nM; D_5_, K_i_ ≈ 228 nM) [66]. Our results showed that non-medicated and patients treated with risperidone exhibited a significant increase in D_3_ expression compared to HV (DN, 15.3%; DF, 9.6%; R, 9.1%). Other studies have reported similar results, showing an increase in D_3_ expression in non-medicated patients in peripheral blood lymphocytes of PWS compared to HV (an increase of 2.3 fold [15], 9.2 fold, [21], 20% [67], 88.5% [68]). In contrast, other studies have reported that D_3_ expression is decreased in PBL or leukocytes in non-medicated patients compared to HV, and that antipsychotic treatment increases D_3_ expression after 6 weeks compared to baseline [69,70]. The discrepancies between our results and those reported by other studies could be due to some differences in the cell isolation procedure or by the consumption of different pharmacological treatments, among other factors.

Although our data did not detect associations between symptomatology and D_3_ expression in PWS, some studies have reported that PWS with the highest D_3_ expression in PBL or granulocytes have a high Brief Psychiatry Rating Scale (BPRS) score [21,71], while D_3_ expression in unmedicated PWS correlates with negative symptoms [69]. There are few studies examining the effect of a specific antipsychotic on D_3_ expression in PBMCs from PWS. However, evidence indicates that clozapine consumption increases D_3_ mRNA levels in CD4 T cells of patients compared to HV [27]. As for the effect of risperidone on D_3_ expression, the evidence demonstrates that PBMCs from medicated patients or cells incubated with risperidone in vitro do not show changes in D_3_ expression when compared with HV [72].

The altered expression of D_3_ in PBMCs from PWS is relevant because this receptor shows the highest affinity to DA (Ki ≈ 27 nM) [66]. Additionally, D_3_ is involved in various cellular functions during the inflammatory response, such as T cells differentiation and the suppression of Treg and mast cells in inflammatory animal models [66,73,74]. The presented data suggests that PBMCs exhibiting heightened D_3_ expression may exhibit anomalous responsiveness following receptor activation by one of its ligands, such as DA, culminating in immune process alteration. The elevated levels of D_3_ in the patients did not decrease with the consumption of risperidone, although this drug may directly alter cell function when it interacts with D_3_. While it has not been precisely shown that the effect is due to D_3_ activation, there is a well described high affinity between D_3_ and risperidone (Ki ≈ 18 nM) [18]. The interaction between the overexpressed D_3_ receptor on leukocytes and DA of PWS with no treatment might overstimulate the cell and modify the immune response. However, risperidone could also stimulate the overexpressed D_3_ receptor, altering cell function and inducing an inflammatory response. Due to inconsistencies in D_3_ expression in peripheral cells, this receptor may not be a suitable marker for schizophrenia. 

Our results indicated that there were no alterations in D_5_ mRNA levels among drug naïve, drug free, or patients treated with risperidone in comparison to HV. Additionally, a study confirmed that D_5_ mRNA levels of peripheral lymphocytes were unchanged among drug-medicated, drug-naïve, and drug-free PWS and HV [21]. Currently, there is no evidence evaluating the impact of risperidone consumption on D_5_ expression in PBMCs of PWS. D_5_ is the second receptor with a higher affinity to DA (Ki ≈ 228 nM). Additionally, reports in mice show that D_5_ stimulation reinforced T-cell activation [66], and the stimulation of these receptors on dendritic cells modulates the differentiation of T CD4^+^ cells towards the Th17 phenotype, showing that D_5_ is involved in cellular activation during the inflammatory response [75]. Other studies have shown that the antagonism of D1-like receptors (both D_1_ and D_5_) inhibits Th17 differentiation in experimental autoimmune encephalomyelitis (EAE) [76], and the agonism of these receptors on human PBMCs impairs CD8^+^ Treg differentiation and activity. However, these studies employed molecules that agonize or antagonize both D_1_ and D_5_ receptors due to the lack of specific drugs that stimulate only D_5_. Therefore, observed effects cannot be specifically associated with the D_5_ stimulation [76,77]. It appears that D_5_ expression in PBMCs is only minimally affected by schizophrenia or the consumption of risperidone. 

Reports evaluating the expression of DAT in leukocytes are limited. Our study revealed increased DAT mRNA levels in all three patient groups compared to HV (DF,16.1%; DN. 12.9%; R, 11.1%), whereas other studies have reported decreased density of DAT proteins (53.8%) in resting lymphocytes of psychotic patients and no significant changes in DAT mRNA levels in non-medicated PBL patients compared to HV [78,79]. It is worth noting that patients with chronic schizophrenia (illness duration greater than two years) overexpress this transporter (48.7%) [79]. DAT is involved in the immune response by regulating the uptake and release of DA in the microenvironment of leukocytes [80]. Studies in mice models demonstrate that DAT expression is required for T CD8^+^ cells and memory B cells’ expansion or survival. Furthermore, myeloid cells with a DAT deletion show a reduction in IL-23 and GM-CSF serum levels, spleen hypoplasia, and promotes a pro-inflammatory phenotype in myeloid cells and macrophages [80]. 

A possible explanation for the increased expression of DAT in PWS, as observed in this study, could be linked to levels of D_3_ and DA in serum. The mechanism involving these three molecules is observed in CNS. An excess release of DA activates D_3_ autoreceptors in neurons, which results in the inhibition of DA synthesis or release [81]. Moreover, in mice, prolonged D_3_ stimulation with agonists leads to DAT degradation [82]. This mechanism may be replicated in the periphery, and DAT expression may be regulated by the stimulation of D_3_ in PBMCs caused by serum DA levels. Experimental evidence shows elevated levels of DA in the serum of PWS compared to HV [83]. This raises the question of whether our results could possibly be explained by this mechanism, where high levels of DA in the serum may lead to the observed permanent elevation of mRNA levels of DAT in patients. This could be due to the cell’s need to synthesize more DAT proteins in response to degradation. Furthermore, there is no evidence that the consumption of risperidone affects this potential mechanism, as patients continue to exhibit increased DAT expression throughout the 40-week pharmacological treatment. Our hypothesis concurs with Marazziti et al.’s report of low DAT protein levels in lymphocytes of drug-free patients and Liu et al.’s finding of increased DAT mRNA levels in PBL of chronic patients. The decrease in DAT proteins may be due to degradation, while the elevation in DAT mRNA levels may be linked to the cells’ requirement for DAT.

The antipsychotic effects resulting from 5-HT receptor antagonists and the evidence of changes in the serotoninergic system in the CNS of PWS support the involvement of 5-HT in the disease’s pathophysiology [84]. Furthermore, the positive outcomes observed in clinical trials utilizing a combination of antipsychotic and selective serotonin receptor inhibitor (SSRI) antidepressant treatments have prompted additional research on the role of the serotoninergic system in this illness, including examining the status of serotoninergic components in peripheral cells. 

In this work, we found relevant and significant results about 5-HT_2A_. PWS without treatment showed an increased expression of 5-HT_2A_ in PBMCs compared to HV (DN, 9.8%; DF, 15.1%). Risperidone consumption resulted in a notable reduction in the expression of this receptor compared to drug-naïve (11.5%) and drug-free (15.6%) groups, even at HV levels. Additionally, the levels of 5-HT_2A_ mRNA indicated a robust correlation with both the PANSS total and positive subscale, alongside a significant negative correlation with the duration of risperidone consumption. Our findings are consistent with reports analyzing this receptor in peripheral cells. Studies suggest that 5-HT_2A_ binding sites are higher in non-medicated PWS compared with HV, and antipsychotic consumption decreases the binding sites during the first 12 months of therapy, However, after the first year of use, 5-HT_2A_ binding sites tend to increase again [85]. Other studies show that 5-HT_2A_ binding sites are higher in nonmedicated patients compared to HV (11.3%), and the antipsychotic consumption increases the expression compared to basal levels (14%) [86]. The finding above implies that antipsychotic treatment has a directly or indirectly impact on the expression of 5-HT_2A_ in peripheral cells. Additionally, studies demonstrate an elevation in 5-HT_2A_ mRNA levels in peripheral blood cells of drug-naïve and drug-free patients with gender disparities [87].

5-HT_2A_ stimulation triggers cellular and systemic effects during an immune response. According to reports, in vitro stimulation with a 5-HT_2A_ agonist [(R)-1-(2,5-dimethoxy-4-iodophenyl)-2-aminopropane, (R)-DOI)] boosts the activation of T CD8^+^ and Th1 cells in mice, while the peripheral stimulation hinders the systemic effects of TNF-α in whole animals, resulting in anti-inflammatory effects, including the inhibition of adhesion molecules, cytokines, and chemokines genes [88]. Furthermore, it has been found that in vitro, the production of IL-2 and IFN-γ by T CD8^+^ cells are decreased in the presence of a 5-HT_2A_ antagonist (sarpogrelate) [89]. Changes in leukocyte 5-HT_2A_ expression may alter the response to 5-HT, potentially contributing to dysfunctional immune mechanisms or clinical symptoms in PWS who have not received treatment. This is significant as 5-HT is a pro-inflammatory molecule in the immune response [12]. 

In this work, we found that treatment with risperidone reduced 5-HT_2A_ expression, which was directly associated with a decline in positive subscale scores. Risperidone is an antagonist of 5-HT_2A_ with a high affinity (Ki ≈ 0.5 nM), and multiple studies have shown that it produces systemic immune alterations leading to an anti-inflammatory profile [18]. Risperidone may ameliorate the low-grade systemic inflammation associated with schizophrenia and contribute to clinical improvement, particularly the positive subscale score. The correlation between 5-HT_2A_ expression and the duration of risperidone consumption demonstrated in this work suggests that 5-HT_2A_ mRNA levels in PBMCs could be used as a potential biomarker of the efficacy of risperidone during pharmacological treatment. The regulation of the serotoninergic system is a significant therapeutic target for enhancing the clinical condition of PWS. Antidepressant drugs are commonly administered with antipsychotics to treat the symptoms of schizophrenia, primarily the negative symptoms, leading to improvement [90]. 

5-HT or antipsychotic medications do not directly interact with the serotonin transporter (SERT) in a ligand–transporter manner. However, our results have demonstrated that untreated PWS have increased SERT expression compared to HV (DN,12.7%; DF, 16.8%), and risperidone treatment decreased the expression of this transporter in comparison to drug-naïve (8.4%) and drug-free (11.6%) groups. Furthermore, it was found that SERT mRNA levels were directly correlated with PANSS total score and positive subscale score, with an inverse correlation between SERT expression and weeks of risperidone use. Consistent with our findings, previous studies have reported a greater expression of SERT in PBMCs (and platelets) in nonmedicated PWS compared to HV (107.3%) [91]. Notably, the male population has demonstrated the most significant overexpression of this transporter (190%) [87]. Furthermore, there is evidence of an association between increased SERT activity in lymphocytes and aggressive behavior in PWS [92]. In addition, one report finds that patients with 8 weeks of risperidone consumption decreased SERT expression in peripheral blood cells compared to HV [93]. However, evidence suggests that patients undergoing antipsychotic treatment display a reduction in SERT density in platelets when compared to nonmedicated patients (51.3%) [91]. Also, we did not find data that show correlations between SERT expression and PANSS total or positive subscale scores. However, there is evidence that expose a negative correlation between SERT and negative subscale score in the PBMCs of female drug-free patients [87].

SERT plays a role in the immune response by regulating the uptake and release of 5-HT in cells. Notably, serotonylation, a SERT-dependent mechanism, is required for certain immune functions such as efferocytosis in macrophages [94], migration and cytokine secretion [12]. The levels of 5-HT serum play a crucial role in regulating SERT expression in leukocytes and platelets [95]. Research reveals that an increase in 5-HT in horses’ blood after treadmill exercise leads to a remarkable drop in SERT levels, including protein density and expression in leukocytes and platelets [96]. These findings support the notion that higher 5-HT plasma levels may reduce SERT expression, whereas lower levels may enhance it [97]. In this context, PWS without treatment show a decrease in 5-HT serum levels compared to HV, and the use of clozapine for eight weeks increases 5-HT concentrations compared to baseline [98]. 

The elevated expression of SERT in nonmedicated patients depicted in this study may be related to decreased serum 5-HT levels, as reported by Ertugrul et al. Moreover, our findings revealed a substantial reduction in SERT expression due to risperidone consumption, plausibly linked to augmented serum 5-HT levels following drug intake. Based on our findings and previously available evidence, we suggest that risperidone could potentially elevate 5-HT levels in serum patients via an unestablished mechanism and concurrently reduce SERT expression in PBMCs. Our results suggest that the consumption duration of risperidone and SERT expression have an inverse relationship, as demonstrated in this study. Measuring 5-HT serum levels is necessary to confirm the potential link between risperidone consumption and peripheral 5-HT serum levels, as well as the association with SERT expression in the PBMCs of patients. Our findings suggest that SERT expression could serve as a peripheral marker for monitoring the clinical outcomes of risperidone treatment. 

It Is well established that the dopaminergic and serotonergic neurotransmission is altered in PWS, which can lead to psychotic symptoms [99]. These alterations are linked to the concentration of neurotransmitters and the number of receptors found in neurons in specific regions of the CNS [9]. Our research shows that nonmedicated PWS have increased expression of DA and 5-HT receptors and transporters in PBMCs, while those taking risperidone exhibit a decrease in 5-HT_2A_ and SERT mRNA levels. These findings suggest that could be an association between alterations in DA and 5-HT blood levels and the observed changes. The evidence suggests that PWS without treatment have high serum levels of DA and low serum levels of 5-HT [83,98]. Subsequently, we posit that the intake of risperidone could augment the levels of 5-HT in serum, consequently leading to an alteration in the expression of SERT and 5-HT_2A_ as an indirect outcome of pharmacological intervention. However, risperidone does not alter serum levels of DA, nor does the drug’s effect sufficiently alter expression of D_3_ and DAT, which results in the maintenance of high mRNA levels of these molecules by the patients. Further research on the effects of antipsychotics on the peripheral concentration of DA and 5-HT in PWS is required.

The physiopathology of schizophrenia is based on alterations in neurotransmitter circuits, particularly in the dopaminergic neurotransmission [100]. However, other neurotransmitter circuits, such as the serotoninergic, are also linked to this disease [5,101]. This altered phenotype in patients’ brains causes the symptoms of schizophrenia. However, patients also exhibit altered phenotypes in other peripheral cells due to the presence of the disease, such as phenotypic changes in leukocytes and abnormal levels of humoral molecules, including the dopaminergic and serotoninergic circuits (receptors, transporters, and dopamine and serotonin serum levels) [14,83,98]. 

These changes in peripheral leukocytes and humoral molecules can lead to alterations in cellular response. This supports the presence of a low-grade inflammation state in patients with schizophrenia [102], characterized by the increase in inflammatory molecules like cytokines [103]. The inflammatory dysregulation contributes to the severity of the clinical state and symptomatology [104].

Antipsychotic treatment acts on CNS cells and improves brain function, thereby reducing symptomatology [105]. However, these drugs also cause leukocyte changes and modify the already altered phenotype in patients. For example, we have shown that risperidone reduces the increased expressions of 5-HT_2A_ and SERT in the PBMCs of patients. Based on the evidence, antipsychotic drugs, especially atypical drugs, decrease the inflammatory state of patients, indirectly reducing symptoms. The abovementioned bidirectional interaction is an example of the complex neuro-immuno-endocrine supersystem that enables optimal organism functioning. Alterations in one system may modify the others, leading to organic changes that contribute to the development of pathologies.

The psychiatric field could benefit from the use of molecular tools for the diagnosis and clinical follow up of PWS [68]. The use of blood instead of other biological samples or imaging studies has advantages such as accessible sample collection, less invasiveness, a more straightforward procedure, and lower cost. In addition, the analysis of receptors and transporters of DA and 5-HT in peripheral blood cells is a useful molecular tool to evaluate the changes and functional properties of receptors and transporters that occur during the development of schizophrenia or during antipsychotic use, even if these changes do not reflect those observed in the brain. Our work has found strong evidence in the current literature to support our findings. However, we also describe studies that are inconsistent with our data. The main conflict we found with the reported evidence is that there is great heterogeneity in the inclusion and exclusion criteria in studies such as this one. It is necessary to carry out studies with stricter criteria, as well as meta-analysis studies that analyze the data already reported, in order to reach conclusions that help us deepen our knowledge of the pathophysiology of this disorder and a better therapeutic strategy for patients. 

The significance of our work lies in presenting information that demonstrates the systemic effects resulting from antipsychotic consumption. While the primary goal of antipsychotic treatment is to alleviate symptoms during a patient’s psychotic state, it is important for psychiatrists to be aware that these drugs also have peripheral effects and can disrupt the function of other systems in the body, including the immune system. Our aim is to provide new research on the peripheral effects of psychiatric treatments, allowing psychiatrists to choose the optimal treatment for each patient.

Additionally, our work contributes to the clinical field by providing evidence of the potential use of two quantitative parameters to evaluate treatment with risperidone. There are currently no quantitative parameters available to support psychiatrists in the diagnosis or clinical follow-up of schizophrenia. Therefore, the search for quantitative parameters is necessary to aid clinical psychiatry. This study suggests that the measurement of 5-HT_2A_ and SERT expression could serve as possible markers to determine the efficacy of treatment with risperidone. However, further studies are needed to replicate these results.

This study has limitations that need to be considered. First, the restrictive inclusion criteria resulted in a smaller sample size. Second, drug-free patients did not truthfully report when they stopped their last antipsychotic treatment, so this variable was not reported in this study. In addition, we only used one reference gene in this study: β-actin gene *ACTB.* Finally, drug-free patients may have used another type of psychoactive drug as part of their previous pharmacological treatment, which was not included as a variable in this study. Multicenter studies with larger sample sizes are needed to provide solidity, reproducibility, and consistency to the findings reported in this work.

## 4. Materials and Methods

### 4.1. Participant Information and Clinical Measures 

We recruited 218 PWS from August 2015 to March 2020 in the general outpatient services of Instituto Nacional de Psiquiatría Ramón de la Fuente Muñíz, Mexico. The Ethics Committee at Instituto Nacional de Psiquiatría Ramón de la Fuente Muñíz approved the study with the number SC-1524-14. Fifty-six patients met the inclusion criteria. Inclusion criteria for the study were as follows: male and female participants aged between 18 and 55 who were diagnosed with schizophrenia based on the Diagnostic and Statistical Manual of Mental Disorders, fifth edition (DSM-V) diagnostic criteria. The participants were categorized into three groups, which are drug-naïve patients (DN), drug-free patients (DF; who stopped taking antipsychotic drugs for at least four weeks before participation), and patients with pharmacological treatment with risperidone for a maximum of forty weeks (R). Exclusion criteria included intellectual disability, the presence of acute or chronic infectious and severe physical diseases, state of pregnancy, thyroid, autoimmune or malignant hemato-oncological diseases, uncontrolled metabolic diseases, the presence of any immune hypersensitivity, and immunomodulator treatment consumption. The participants received a complete and detailed explanation of the objectives of the study. Signed consents were obtained from all participants before the beginning of the study. The severity of symptoms was assessed using the Spanish version of the positive and negative syndrome scale (PANSS), whose five component scores were used to analyze the different domains [106]. The control group consisted of HV with a negative history of psychiatric or neurological disorders who did not have any history of neurological or psychiatric disorders, nor any current acute inflammatory or infectious conditions. Table 1 presents the participants’ demographic data. 

### 4.2. Sample Processing

Venous whole blood samples were collected from all participants by venipuncture and placed in tubes containing preservative-free heparin (BD Vacutainer; Franklin Lakes, NJ, USA; No. 367878). PBMC isolation was performed within 2 h after blood collection until the cDNA synthesis. The PBMCs were purified by centrifugation (461 g for 30 min) using a Ficoll-Paque density gradient (Sigma-Aldrich, St Louis, MO, USA; No. 10771). The cells were washed twice in phosphate-buffered saline (PBS) and lysed in TRIzol solution (Invitrogen Life Technologies, Carlsbad, CA, USA; No. 15596-018) for RNA extraction according to the manufacturer’s instructions. The quality and concentration of the extracted RNA was measured using a NanoDrop spectrophotometer (Thermo Scientific, Waltham, MA, USA; model 2000/2000c), while the integrity was assessed by gel electrophoresis. The RNA was treated with DNAse (Invitrogen Life Technologies, Carlsbad, CA, USA) to remove any possible genomic DNA contamination. Reverse transcription to obtain cDNA was performed in a thermocycler Gradient Thermocycler; (Eppendrof, Hamburg, Germany, model 5531) using a 1 μg RNA. 

cDNA samples were analyzed weekly to obtain qPCR results. The qPCR was performed (by duplicate) in 5 μL of a reaction mixture containing 2.5 μL buffer TaqMan Universal PCR Master Mix (Applied Biosystems, Foster City, CA, USA; No. 4304437), 25 ng (0.5 μL) cDNA, 1.75 μL nuclease-free water, and 0.25 μL of TaqMan^TM^ probe (Foster City, CA, USA; *DRD3*, Hs00364455_m1; *DRD5*, Hs00361234_s1; *SLC6A3*, Hs00997374_m1; *HTR2A*, Hs01033524_m1; *SLC6A4*, Hs00169010_m1; *ACTB*, Hs01060665_g1; No. 4331182) on a thermocycler (CFX96 Real-Time System, Bio-Rad Laboratories Inc., Hercules, CA, USA). The PCR cycler consisted of an initial incubation at 95 °C for 10 min, 40 cycles of denaturation at 95 °C for 15 s, and annealing/extension at 60 °C for 1 min. The Cq values were estimated by analyzing each sample using the CFX Manager Software v3.1 (Bio-Rad Laboratories Inc., Hercules, CA, USA). This software is a program designed for an intuitive experiment setup and data analysis with the CFX96 Real-Time System. The relative expression of each gene was normalized to β-actin expression as follows: Cq target gene/Cq β-actin.

### 4.3. Statistical Analysis 

The statistical analysis for all data was performed using GraphPad Prism 10.0.3 software (San Diego, CA, USA). To determine a Gaussian distribution, a Shapiro–Wilk normality test was used, and based on the obtained results, the following tests were performed. To evaluate differences in the BMI, mRNA levels of D_3_, DAT, 5-HT_2A_, SERT, and PANSS total and subscales scores from patients we applied the one-way ANOVA (F) and Tukey post hoc tests. Kruskal–Wallis (H) and Dunn’s post hoc tests were used to analyze the age; the excitability subscale scores; and D_5_ mRNA levels. We evaluated the correlation between gene expression and clinimetric scores as well as the duration of risperidone consumption in PWS. Pearson correlation test (*r*) was used to assess the correlation of the following pairs: D_3_ vs. positive subscale; D_3_ vs. negative subscale; D_3_ vs. cognitive subscale; DAT vs. PANSS total scale; DAT vs. cognitive subscale; 5-HT_2A_ vs. PANSS total scale; 5-HT_2A_ vs. positive subscale; 5-HT_2A_ vs. negative subscale; 5-HT_2A_ vs. cognitive subscale. For the remaining pairs we assessed them using Spearman’s rank correlation test (*r_s_*). A statistically significant difference was recognized when *p* < 0.05. A moderated correlation was recognized when 0.4 *< r* or *r_s_ <* 0.6, and a strong correlation was recognized when 0.6 *< r* or *r_s_ <* 0.9 [107].

## 5. Conclusions

This work reinforces the notion that serotonergic alterations may be associated with the pathophysiology of schizophrenia, and we demonstrated that risperidone consumption exhibits a direct effect on 5-HT_2A_ and SERT expression. Furthermore, we have shown that risperidone treatment does not alter DA receptors and DA transporter, thus patients maintain high mRNA levels of D_3_ and DAT despite antipsychotic treatment. The expression of 5-HT_2A_ and SERT was directly correlated with PANSS total and positive subscale scores, and finally, their expression was inversely correlated with the weeks of risperidone consumption. We hypothesized that receptor and transporter expression may be associated with serum levels of DA and 5-HT in patients, and 5-HT_2A_ and SERT expression in PBMCs may be potential treatment response biomarkers for clinical follow-up of risperidone-treated PWS. Although the main objective of antipsychotic treatment is the reduction in symptoms during the patient’s psychotic state, it is imperative that treating psychiatrists are aware that these drugs also cause peripheral effects and alter the function of other systems in the organism, such as the immune system. We will continue exploring the effect of risperidone monotherapy on inflammatory markers such as cytokines, the number of leukocyte subtypes, and other relevant molecules such as serotonin serum levels. In addition, we will evaluate the effect of other antipsychotic drugs monotherapy consumption in patients with schizophrenia. 

## Figures and Tables

**Table 1:** Demographic parameters and PANSS scale and subscale scores of healthy volunteers and patients with schizophrenia.

Parameter	HV	DN	DF	R	F or H Value	*p*
n	10	11	17	28	-	-
Age ^b^	27.30 ± 4.71	33.27 ± 13.86	38.24 ± 12.15	28.00 ± 8.74	H (3) = 7.751	0.0514
BMI ^a^	24.02 ± 2.58	26.22 ± 3.22	25.44 ± 3.31	25.73 ± 3.18	F (3, 62) = 0.982	0.4069
Gender female: male	4: 6	4: 7	6: 11	9: 19	-	-
Smoke yes: no	1: 9	4: 7	7: 10	10: 18	-	-
PANSS total and subscale scores						
TOTAL ^a^	-	85.73 ± 20.30	95.47 ± 19.45	73.36 ± 17.46	F (2, 53) = 7.652	0.0012
Positive subscale ^a^	-	29.18 ± 6.60	30.94 ± 7.80	18.75 ±7.30	F (2, 53) = 17.410	<0.0001
Negative subscale ^a^	-	21.55 ± 9.79	24.00 ± 6.48	22.04 ±7.57	F (2, 53) = 0.452	0.6388
Cognitive subscale ^a^	-	17.27 ± 4.92	19.94 ± 4.02	16.46 ± 4.49	F (2, 53) = 0.077	0.9254
Excitability subscale ^b^	-	8.09 ± 3.98	9.52 ± 3.31	6.75 ± 3.26	H (2) = 6.791	0.0335
Depression subscale ^a^	-	9.56 ± 4.56	11.06 ± 3.45	9.35 ± 3.64	F (2, 53) = 1.112	0.3365

HV, healthy volunteers; DN, drug-naïve patients; DF, drug-free patients; R, patients treated with risperidone; BMI, body mass index. Mean ± SD. ^a^ One-way ANOVA test, Tukey post hoc test. ^b^ Kruskal–Wallis test, Dunn’s post hoc test.

**Table 2:** Correlation data of PANSS total and subscale scores with mRNA levels of D_3_, D_5_, DAT, 5-HT_2A_, and SERT in patients with schizophrenia.

	Receptor or Transporter	D_3_	D_5_	DAT	5-HT_2A_	SERT
Scale/ Subscale Score						
PANSS total		r_s_ = −0.1501 *p* = 0.2785	r_s_ = −0.1611 *p* = 0.2447	r = −0.1352 *p* = 0.3297	r = 0.5801 *p* < 0.0001	r_s_ = 0.5199 *p* < 0.0001
Positive		r = −0.1306 *p* = 0.2465	r_s_ = −0.1258 *p* = 0.3647	r_s_ = 0.0010 *p* = 0.9939	r = 0.5608 *p* < 0.0001	r_s_ = 0.5698 *p* < 0.0001
Negative		r = −0.0200 *p* = 0.8858	r_s_ = −0.0533 *p* = 0.7178	r_s_ = −0.1353 *p* = 0.3293	r = 0.2426 *p* = 0.0772	r_s_ = −0.0409 *p* = 0.7689
Cognitive		r = −0.2433 *p* = 0.0763	r_s_ = −0.1106 *p* = 0.4258	r = −0.1581 *p* = 0.2534	r = 0.0364 *p* = 0.0073	r_s_ = 0.2578 *p* = 0.0598
Excitability		r_s_ = −0.0763 *p* = 0.5833	r_s_ = −0.1037 *p* = 0.4554	r_s_ = −0.2180 *p* = 0.1133	r_s_ = 0.2346 *p* = 0.0878	r_s_ = 0.2057 *p* = 0.1356
Depression		r_s_ = −0.1877 *p* = 0.1742	r_s_ = −0.1659 *p* = 0.2306	r_s_ = −0.0332 *p* = 0.8113	r_s_ = 0.1680 *p* = 0.2245	r_s_ = 0.3679 *p* = 0.0067

r, Pearson correlation test; r_s,_ Spearman’s rank correlation test.

**Table 3:** Characteristics of DA and 5-HT receptors and transporters and evidence of changes in central nervous system.

Receptor or Transporter	Family	Action Mechanism	Function	Distribution in CNS	Distribution in Leukocytes	Examples of Reported Alterations in PWS
D_3_	D_2_-like subfamily [28].	It is coupled to the G_ai/o_ protein. It inhibits AC, blunts cAMP formation, and PKA activation [28].	It modulates rewarding and motivating behavior, some features of cognitive functions, and locomotor activity [29].	The ventral striatum (nucleus accumbens), thalamus, hippocampus, islands of Calleja, cerebellum, cortex, substantia nigra, and VTA [30].	T and B lymphocytes, dendritic cells, neutrophils, monocytes, and NK cells [13].	The increase in D_3_ levels in the rostral and caudal basal ganglia structures of drug-free patients. The decrease in the expression in the parietal and motor cortices of medicated chronic patients [31,32].
D_5_	D_1_-like subfamily [33].	Coupled to G_a2_/G_olf_ protein that initiates the cAMP/PKA signaling via AC activation [33].	Involves in working memory, visual attention, and recency memory [34].	Cortical, subcortical, and limbic regions such as the cerebral cortex. Hippocampus, substantia nigra pars compacta, hypothalamus, striatum, nucleus accumbens, cerebellum, and olfactory tubercule [35].	Dendritic cells, T and B lymphocytes, eosinophils, neutrophils, NK cells, and platelets [13,36].	Reduction in D_1_-like receptors in PFC of drug-naïve and drug-free patients [7]. A decreased density in basal ganglia of drug-naïve patients [37].
DAT	SLC6 class of NA^+^/Cl^−^ dependent transporters [38].	It reuptakes DA from the synaptic cleft back inside the presynaptic neuron for a rerelease, limiting DA effects [39].	The principal regulator of DA neurotransmitter synaptic availability in mammals [39].	DA neurons—high density in the striatum and nucleus accumbens-, anterior cingulate, amygdala, midbrain, and frontal areas [40].	B and T lymphocytes, macrophages, and platelets [13].	No DAT expression changes in the striatum of non-medicated patients [41]. A decreased DAT expression in the striatum in naïve patients [42]. A decreased expression in the striatum [43], amygdala [44], and an increased expression in basal ganglia [45] and Brodmann area 9 [43] in drug-treated patients.
5-TH_2A_	Serotonin receptor family [46].	It is involved in the regulation of the mood, and acts as a cognitive process modulator, such as memory and learning [47].	It is the primary excitatory 5-HT receptor in the brain [46].	High density: the dendrites of layer V cortical pyramidal glutamatergic cells [46]. Intermediate density: hippocampus, basal ganglia, thalamus, putamen, and nucleus accumbens [48]. Minimal density: cerebellum and brain stem [48].	B and T lymphocytes, monocytes and macrophages, dendritic cells, eosinophils, and platelets [12].	A decrease in 5-HT_2A_ receptors in the caudate nucleus [6] and PFC [49] of drug-naïve patients and individuals with an elevated risk. Increased 5-HT_2A_ binding sites in PFC in antipsychotic free patients but no in medicated patients [50]. Medicated patients with decreased density in the superior temporal cortex [51], BA9 [52], superior temporal gyrus [53], and lower mRNA expression in the hippocampus [54].
SERT	SLC6 class of NA^+^/Cl^−^ dependent transporters [55].	It transports 5-HT from the synaptic cleft to the presynaptic neuron [56].	It regulates the duration and concentration of 5-HT in the synaptic cleft via a saturable reuptake mechanism [56].	Serotonergic neurons that project from raphe nuclei of the pons and upper brain stem to the hypothalamus, thalamus, amygdala, striatum, and cortical mantle [57,58].	Platelets, T and B lymphocytes, dendritic cells, mast cells, and monocytes [12].	The increase in SERT density in the caudate nucleus, nucleus accumbens, dorsal putamen [59], frontal cortex (BA9), and a decreased density in the temporal cortex (BA21) [60] in patients.

AC, adenylyl cyclase; cAMP, cyclic adenosine monophosphate; PKA, protein kinase A; VTA, ventral tegmental area; PFC, prefrontal cortex; 5-HT, serotonin; DA, dopamine; BA, Brodmann area; NA^+^, sodium ion; Cl^−^, chloride ion; SLC6, solute carrier family 6; NK, natural killer.

## Data Availability

The data presented in this study are available on request from the corresponding author. The data are not publicly available due to the data protection law, Mexico.
